# Supplementary material for: Previous exposure to dengue virus is associated with increased Zika virus burden at the maternal-fetal interface in rhesus macaques
Source: PLoS Negl Trop Dis. 2021 Jul 30;15(7):e0009641. doi: 10.1371/journal.pntd.0009641 (PMC8357128; doi:10.1371/journal.pntd.0009641)
Supplement: S1 Fig — PRNT titers against DENV (A-C) and ZIKV (D-E) at 28 days post-DENV challenge, 0 days post-ZIKV challenge, and 28–35 days post-ZIKV challenge. (PDF) [file pntd.0009641.s001.pdf]

Supporting Information

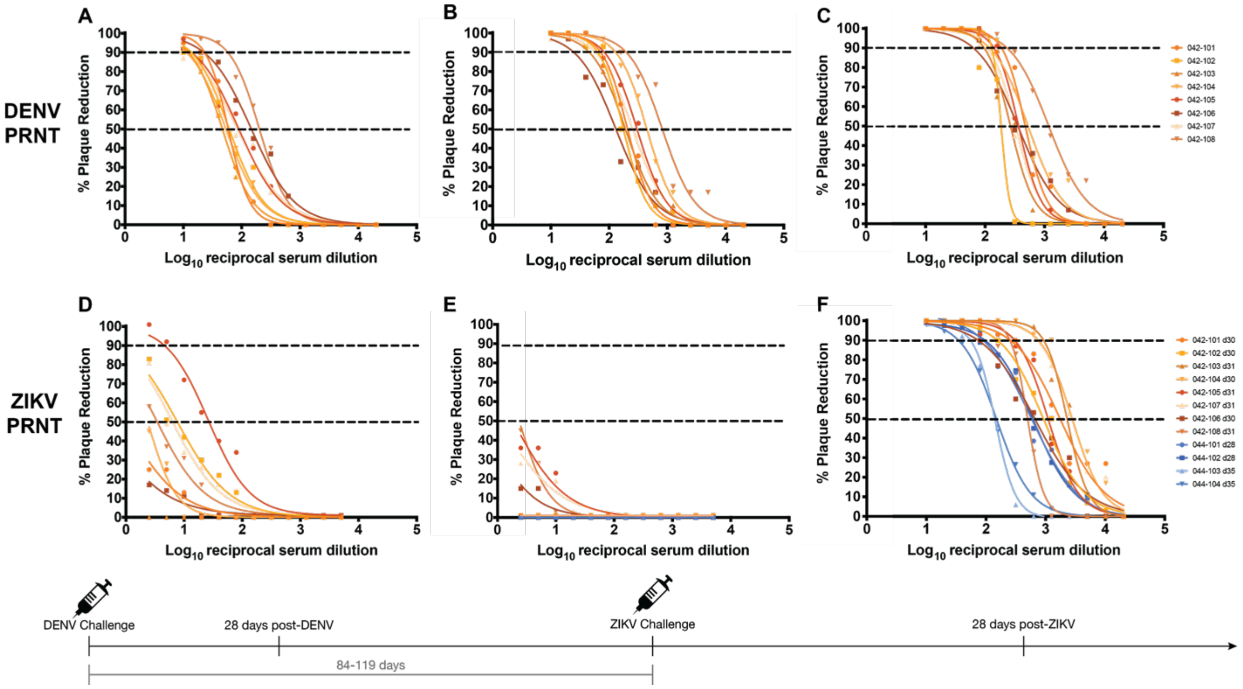

S1 Fig. PRNT neutralization curves. PRNT titers against DENV (**A-C**) and ZIKV (**D-E**) at 28 days post-DENV challenge, 0 days post-ZIKV challenge, and 28-35 days post ZIKV challenge.
