# Supplementary material for: Previous exposure to dengue virus is associated with increased Zika virus burden at the maternal-fetal interface in rhesus macaques
Source: PLoS Negl Trop Dis. 2021 Jul 30;15(7):e0009641. doi: 10.1371/journal.pntd.0009641 (PMC8357128; doi:10.1371/journal.pntd.0009641)
Supplement: S2 Fig — An IgM ELISA using ZIKV NS1 antigen was performed on fetal samples taken on the day of delivery. Maternal plasma and serum samples from 042–108 were included as positive (14 d.p.i.) and negative (4 d.p.i.) controls. Internal assay positive and negative controls fell within the expected range defined by the manufacturer. Fetal serum was used when available. In the absence of available fetal serum, fetal plasma was used for 042–502 and umbilical cord plasma was used for 042–501. (PDF) [file pntd.0009641.s002.pdf]

Supporting Information

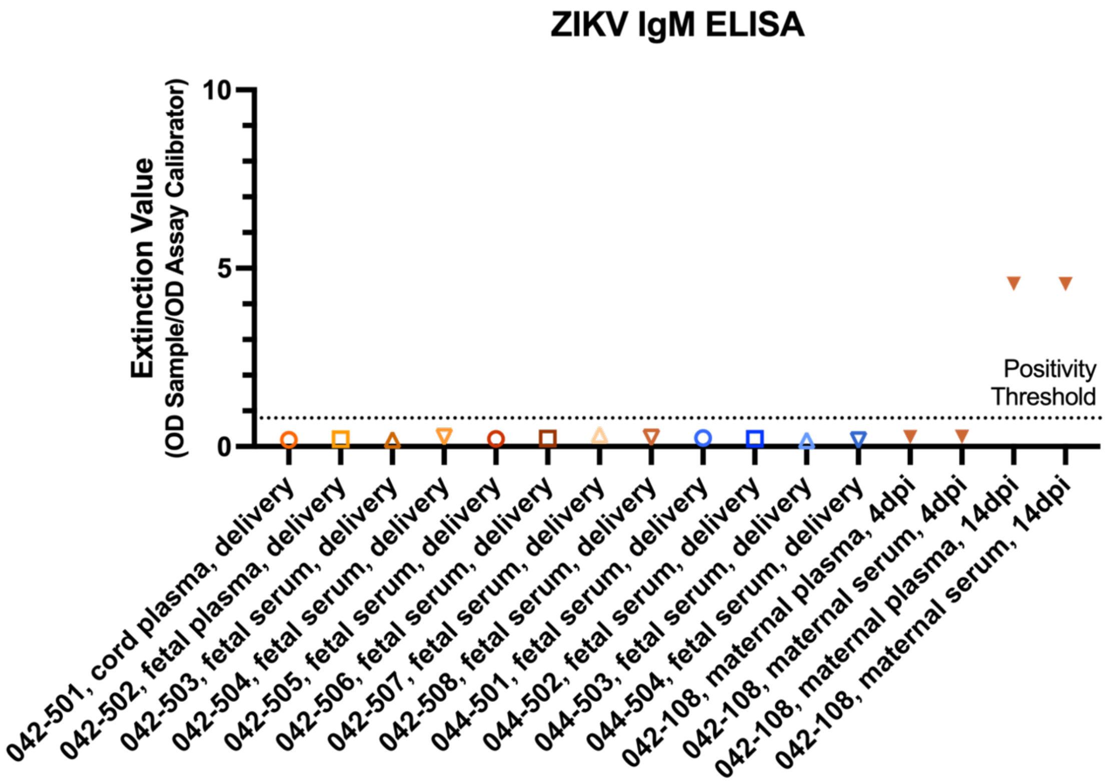

S2 Fig. ZIKV IgM ELISA titers. An IgM ELISA using ZIKV NS1 antigen was performed on fetal samples taken on the day of delivery. Maternal plasma and serum samples from 042-108 were included as positive (14 d.p.i.) and negative (4 d.p.i.) controls. Internal assay positive and negative controls fell within the expected range defined by the manufacturer. Fetal serum was used when available. In the absence of available fetal serum, fetal plasma was used for 042-502 and umbilical cord plasma was used for 042-501.
