# Supplementary material for: Previous exposure to dengue virus is associated with increased Zika virus burden at the maternal-fetal interface in rhesus macaques
Source: PLoS Negl Trop Dis. 2021 Jul 30;15(7):e0009641. doi: 10.1371/journal.pntd.0009641 (PMC8357128; doi:10.1371/journal.pntd.0009641)
Supplement: S3 Fig — The central cross-section of each placental disc was evaluated for 22 pathologic changes. A description of the scoring system can be found in S2 Table. Features specific to the fetal membranes or uterus are noted in disc 1 scoring. Statistical pairwise comparisons between each group were performed for each feature. For quantitative features (1–9, 12–16, 22) a non-parametric Wilcoxon rank sum test was used; for binary features (10–11, 17–21) Fisher’s exact test was used. For quantitative features, the median value is shown with error bars representing the interquartile range. When compared to the mock-infected cohort, the DENV-immune macaques had significantly higher scores for transmural infarction (disc 1: p = 0.0371; disc 2: not significant), chronic villitis (disc 1: p = 0.0207; disc 2: p = 0.0151), avascular villi (disc 1: p = 0.0152; disc 2: not significant), and chronic retroplacental hemorrhage (disc 1: p = 0.0152; disc 2: not significant). (PDF) [file pntd.0009641.s003.pdf]

Supporting Information

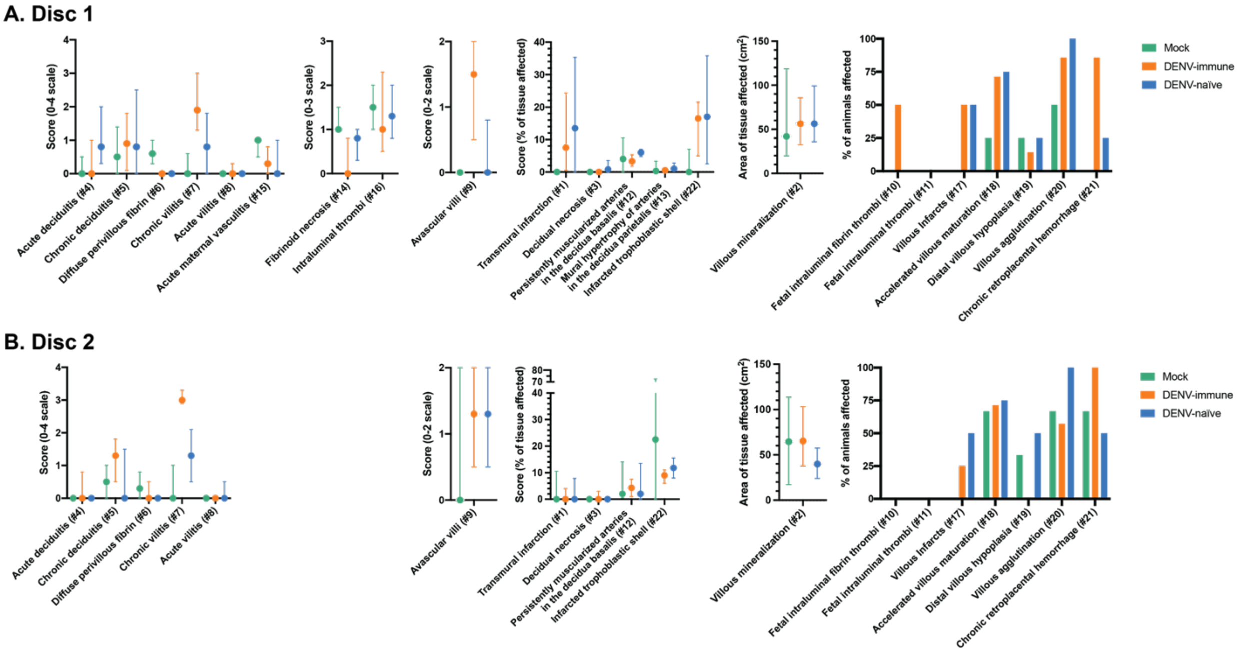

S3 Fig. Placental pathology scoring. The central cross-section of each placental disc was evaluated for 22 pathologic changes. A description of the scoring system can be found in Supplementary Table 4. Features specific to the fetal membranes or uterus are noted in disc 1 scoring. Statistical pairwise comparisons between each group were performed for each feature. For quantitative features (1-9, 12-16, 22) a non-parametric Wilcoxon rank sum test was used; for binary features (10-11, 17-21) Fisher's exact test was used. For quantitative features, the median value is shown with error bars representing the interquartile range. When compared to the mock-infected cohort, the DENV-immune macaques had significantly higher scores for transmural infarction (disc 1: p=0.0371; disc 2: not significant), chronic villitis (disc 1: p=0.0207; disc 2: p=0.0151), avascular villi (disc 1: p=0.0152; disc 2: not significant), and chronic retroplacental hemorrhage (disc 1: p=0.0152; disc 2: not significant).
