# Supplementary material for: Previous exposure to dengue virus is associated with increased Zika virus burden at the maternal-fetal interface in rhesus macaques
Source: PLoS Negl Trop Dis. 2021 Jul 30;15(7):e0009641. doi: 10.1371/journal.pntd.0009641 (PMC8357128; doi:10.1371/journal.pntd.0009641)
Supplement: S1 Table — (PDF) [file pntd.0009641.s004.pdf]

Supporting Information

S1 Table. Maternal and Fetal Tissue and Fluid ZIKV RNA Detection

Supplementary Table 1. Maternal biopsy and fetal fluids

		# positive/total tested	
	**Tissue**	**DENV-immune**	**DENV-naïve**
**Maternal**	Mesenteric lymph node	0/8	1/4
	Spleen	0/2 (042-101, 042-103 not collected)	not collected
	Liver	0/2 (042-101, 042-103 not collected)	not collected
**Fetal**	Umbilical cord plasma	0/8	0/4
	Fetal plasma	0/8	0/4
	Amniotic fluid	0/7 (042-504 not collected)	0/4

Spleen and liver biopsies were not collected from the majority of dams in order to minimize the size of the c-section incision.
