# Supplementary material for: Previous exposure to dengue virus is associated with increased Zika virus burden at the maternal-fetal interface in rhesus macaques
Source: PLoS Negl Trop Dis. 2021 Jul 30;15(7):e0009641. doi: 10.1371/journal.pntd.0009641 (PMC8357128; doi:10.1371/journal.pntd.0009641)
Supplement: S2 Table — (PDF) [file pntd.0009641.s005.pdf]

Supporting Information

S2 Table. Placental Pathology Scoring System (central section)

Supplementary Table 2. Placental pathology scoring system (central section)

Category	#	Feature	Possible scores
General	1	Transmural infarction	0-100%
	2	Villous mineralization/cm² (20x magnification)	area (/cm²)
	3	Decidual necrosis	0-100%
	4	Acute deciduitis	0-4
	5	Chronic deciduitis	0-4
	6	Diffuse perivillous fibrin	0-4
Villitis	7	Chronic villitis: presence of lymphocytes, macrophages	0-4
	8	Acute villitis: presence of neutrophils	0-4
Fetal vascular malperfusion	9	Avascular villi (high grade = 2; low grade = 1; insignificant = 0)	0-2
	10	Fetal intraluminal fibrin thrombi in the stem villlous, chorionic plate vessel, or umbilical cord (present = 1; absent = 0)	0-1
	11	Fetal intramural thrombi in the stem villlous, chorinic plate vessel, or umbilical cord (present = 1; absent = 0)	0-1
Maternal vascular malperfusion	12	Persistently muscularized arteries in the decidua basalis	0-100%
	13	Mural hypertrophy of fetal membrane arteries in the decidua parietalis	0-100%
	14	Fibrinoid necrosis of vessels in the decidua parietalis, decidua basalis, or trophoblastic shell of the basal plate (present = 1/site; max 3)	0-3
	15	Acute maternal vasculitis in the uterus, decidua parietalis, decidua basalis, or trophoblastic shell of basal plate (present = 1/site; max 4)	0-4
	16	Intraluminal fibrin thrombi in the uterus, decidua parietalis, or decidua basalis (present = 1/site; max 3)	0-3
	17	Villous infarcts (present = 1; absent = 0)	0-1
	18	Accelerated villous maturation (AVM): small villi usually with increased syncytial knots (present = 1; absent = 0)	0-1
	19	Distal villous hypoplasia: villi are the same size as AVM but fewer very small tertiary villi and greater intervillous space indicating chronicity (present = 1; absent = 0)	0-1
	20	Villous agglutination: Maternal vascular malperfusion and/or chronic villitis (present = 1; absent = 0)	0-1
	21	Chronic retroplacental hemorrhage: hemosiderin in basal plate or decidua basalis (present = 1; absent = 0)	0-1
	22	Infarcted trophoblastic shell	0-100%
