# Supplementary material for: Previous exposure to dengue virus is associated with increased Zika virus burden at the maternal-fetal interface in rhesus macaques
Source: PLoS Negl Trop Dis. 2021 Jul 30;15(7):e0009641. doi: 10.1371/journal.pntd.0009641 (PMC8357128; doi:10.1371/journal.pntd.0009641)
Supplement: Attachment — Submitted filename: Response to Reviewers.pdf [file pntd.0009641.s006.pdf]

Reviewer's Responses to Questions

Key Review Criteria Required for Acceptance?
As you describe the new analyses required for acceptance, please consider the following:

Methods
-Are the objectives of the study clearly articulated with a clear testable hypothesis stated?
-Is the study design appropriate to address the stated objectives?
-Is the population clearly described and appropriate for the hypothesis being tested?
-Is the sample size sufficient to ensure adequate power to address the hypothesis being tested?
-Were correct statistical analysis used to support conclusions?
-Are there concerns about ethical or regulatory requirements being met?

Reviewer #1: Although it is an interesting, unprecedented study, it has some important limitations. A small number of animals was enrolled. Only one DENV serotype (DENV-2) was tested. In addition, differences between the periods from DENV inoculation to ZIKV challenge in each individual were observed (up to 35 days).

We agree that the small number of animals enrolled in the experiment is a limitation of the study. A formal sample size calculation was conducted prior to study initiation and documented in the study protocol. The planned sample size was 8 animals per cohort which would have allowed detecting an anticipated moderate effect size of d=1.5 when comparing the primary outcomes between the pre-existing DENV cohort vs. the DENV-naïve control cohort with 80% power at the two-sided 0.05 significance level. However, due to an unforeseen change in the study design for the control animal group, only 4 animals were enrolled in the DENV-naive cohort, resulting in a 60% power level to detect an effect size of d=1.5. In the revised manuscript, we acknowledge this limitation of the study in the Discussion section.

We also acknowledge that only testing one serotype is a limitation of this study. However, testing more than one DENV serotype becomes prohibitively expensive. We did not have sufficient resources to test all 4 serotypes and were concerned that there would only be incremental progress by testing multiple serotypes. Shrinking group sizes to accommodate multiple serotypes would have hindered our ability to discern differences between groups and the impact of serotype on outcome. Therefore, we focused on one serotype, DENV-2. DENV-2 is associated with enhancement and is highly prevalent in the Americas where the most recent ZIKV epidemic occurred {32979816; 24468533}. We have added this to our discussion.

With regard to the duration between DENV and ZIKV infection, this is a necessary limitation of the study design. Because the priority was to challenge animals with ZIKV as closely as

possible to the same (early) day of pregnancy, animals could not be pregnant when they were challenged with DENV. Animals were bred at the same time following DENV challenge, but there was inherent, uncontrollable variability in the time at which the animals actually became pregnant. Despite variability in the time between infection, neither the DENV nor ZIKV PRNT antibody titers at the time of ZIKV challenge were positively correlated with the time between infection ($r^2 < 0.1$).

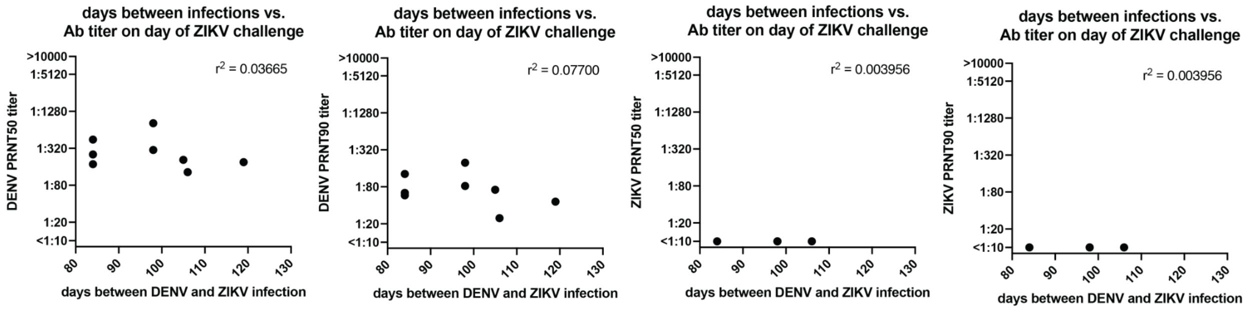

Reviewer #2: Yes, to all of the above questions.

Reviewer #3: Both assays and assay description should be carefully examined here - terms such as "exposure" and "infection" can be interpreted loosely and infection measurements by RNA quantification must be backed up by other experimental results. Additional assays to explain the discrepancy between increased "infection" or viremia at the maternal-fetal interface but no increased "infection" by viral RNA in plasma need to be done.
Both DENV and ZIKV antibodies should be quantified from the offspring and mothers at various timepoints post-birth.

We have revised the manuscript to be precise with our use of “exposure,” “challenge,” and “infection”.

We chose qRT-PCR rather than direct culture for infectious virus because of qRT-PCR’s high throughput and sensitivity. We do know from our previous studies that the PCR titer to PFU ratio for this virus in our hands is ~1:1000.

With regard to our observation of enhanced vRNA burden in the maternal-fetal interface in the absence of enhanced viremia, the immunological mechanism(s) of ADE are not fully understood in heterosubtypic DENV infection and may not perfectly translate to ZIKV. It is conceivable, however, that pre-existing immunity could enhance viral burdens in tissues without necessarily detectably increasing viremia, for example if ADE specifically enhanced infection of FcR-bearing cells in the placenta. Therefore, the lack of enhanced viremia does not preclude the possibility of enhanced tissue burden at the maternal-fetal interface and/or more severe fetal outcomes. We have included this information in our discussion.

We have completed additional IgM ELISAs on fetal serum on the day of delivery from infants to understand the possibility of vertical transmission, reasoning that infection of the fetus would generate an endogenous IgM response, while maternal IgM would not cross into the fetus. Assays detecting IgG, such as ELISA or neutralization tests, could not distinguish between transferred maternal antibody and endogenous fetal responses at this timepoint. The results from these experiments are included in the manuscript (Lines X, Supplementary Figure 2). We have described the implications of these experiments in response to the reviewer's comment in the conclusions section below. As stated, infants from this cohort are undergoing long-term behavioral analysis as part of a different study. Infant blood is being collected for longitudinal antibody analysis, which will be part of a future manuscript.

Reviewer #4: The study objective has been clearly stated. Authors sought to investigate the impact of pre-existing dengue virus immunity on Zika virus infection and pathogenesis during pregnancy by using the nonhuman primate model. The number of animals are small per each group. However, this study utilizes rhesus macaques and involves infections during pregnancy. That can be a constraint on increasing the animals numbers in the study. Regulatory requirements are met for this study.

We agree that the small number of animals enrolled in the experiment is a limitation of the study and appreciate the reviewer's acknowledging the constraints of NHP work. Please see our first comment to Reviewer #1 above.

Results

-Does the analysis presented match the analysis plan?

-Are the results clearly and completely presented?

-Are the figures (Tables, Images) of sufficient quality for clarity?

Reviewer #1: The authors observed a significantly greater burden of ZIKV RNA in the chorionic plate in DENV-immune macaques as compared to DENV-naïve macaques as well as an association between prolonged viremia and the presence of ZIKV vRNA in the maternal-fetal interface. Since previous evidence suggest that the elapsed time from the DENV exposure to the ZIKV infection may influence in the risk of congenital Zika syndrome (Carvalho MS et al. Association of past dengue fever epidemics with the risk of Zika microcephaly at the population level in Brazil. Sci Rep. 2020;10:1752), the comparison between groups of macaques with different elapsed times between DENV and ZIKA infections would be useful.

We agree with the reviewer that risk of vertical transmission and/or of severe complications may vary with the time between DENV exposure and ZIKV infection, however, as stated above, it is beyond the scope and available resources of the present study to conduct an experiment that is powered to address this question. Moreover, as noted above, despite a variability in the time between infection, neither the DENV nor ZIKV PRNT antibody titers were correlated to the time

between infection ($r^2 < 0.1$), suggesting that times between infections were not different enough to significantly affect antibody titers.

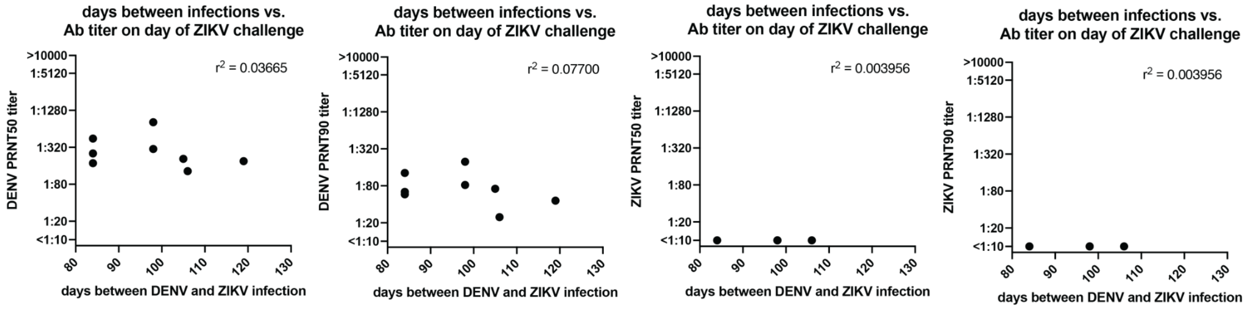

Reviewer #2: Yes, to all of the above questions.

Reviewer #3: Lines 181-192
This paragraph is extremely confusing, to the point of not having a decipherable meaning. First, the word "fell" on Line 181 makes no sense here. The statement that DENV ELISA titers were up 4-fold in 6 of 8 and 4 of 4 animals is not in any context. The statements "cross-reactive ZIKV titers were stable or increased" then followed by "cross-reactive ZIKV antibodies were undetectable in 3 of 4 macaques" are not compatible. This entire section needs to be rewritten in a clear, concise manner so that results can be interpreted and conclusions can be drawn.

This section has been revised for clarity (Lines X-X).

Reviewer #4: The results are presented well. The figures have provided data points for all the animals in the study. Immunological assays for several time points were not accomplished due to COVID-19 limitations.

Conclusions
-Are the conclusions supported by the data presented?
-Are the limitations of analysis clearly described?
-Do the authors discuss how these data can be helpful to advance our understanding of the topic under study?
-Is public health relevance addressed?

Reviewer #1: The work by Crooks et al. entitled "Prior dengue immunity enhances Zika virus infection of the maternal-fetal interface in rhesus macaques" evaluated the impact of prior DENV immunity over the infection of maternal-fetal interface tissues with ZIKV in macaques. This is the first study to assess the aforementioned relationship in a translational non-human primate model.

Reviewer #2: Yes, to all of the above questions.

Reviewer #3: The inability to examine fetal tissues results in the inability to claim "no vertical transmission". There is also no mechanism proposed or examined to explain the disconnect between the increased infection in maternal-fetal interfaces and lack of enhanced viremia in maternal plasma. In addition, other assays are necessary (immune profiling - T cell, innate response, cytokine profiling; disease severity indicators, etc) in order to claim whether there are signs of enhancement or not.

We have completed additional IgM ELISAs on fetal samples on the day of delivery from infants to understand the possibility of vertical transmission. The results from these experiments are included in the manuscript (Lines X, Supplementary Figure 2). We could not detect IgM in any of the fetal serum samples. Because IgM responses wane after infection, we cannot exclude the possibility that vertical transmission occurred earlier in gestation, but these results indicate that there was no detectable immune response to ZIKV in the fetuses at the time of sample collection. We have added QRT-PCR results from fetal plasma taken on the day of delivery. Even testing fetal tissue samples would not allow us to conclusively state whether vertical transmission occurred since it is possible that fetal infection occurred at some point during gestation but was cleared by the time of delivery. Notably, we state throughout that we find no evidence of vertical transmission, but that our assays cannot exclude the possibility that vertical transmission occurred, but was not detected, e.g., because infection of the fetus was short-lived and resolved prior to delivery (Lines X, X, X).

We have expanded the discussion to include consideration of why we may have seen increased infection of the placenta in the absence of viremia differences. We are limited by the small group sizes inherent to non-human primate studies. Because we were able to collect multiple placental samples from each macaque, this increased our power to detect differences in tissue burden in the absence of a difference in viremia. We did correlate prolonged viremia to presence of virus in the MFI, which may be a marker for a process that facilitates enhanced infection of the MFI. One of 4 DENV-naive animals had prolonged viremia and 5 of 8 DENV-immune animals had prolonged viremia. Or perhaps clearance from MFI tissues is delayed relative to clearance of virus from plasma, such that we can detect higher levels of MFI tissue burden remaining at birth in dams who had prolonged viremia. The lack of enhanced viremia does not preclude the possibility of enhanced tissue burden at the maternal-fetal interface and/or more severe fetal outcomes.

The goal of the present study was to determine whether previous DENV exposure increased risk of fetal harm, vertical transmission, and/or infection of maternal-fetal interface tissues, and a comprehensive examination of potential immune mechanisms or markers for such outcomes was beyond its scope. We fully agree with the reviewer that additional studies are required to gain a more comprehensive understanding of the immunological mechanisms of DENV-ZIKV

antigenic interactions and their sequelae. Such studies are underway in our own laboratories (and likely others).

Reviewer #4: Authors need to add sufficient justification about the pathogenic potential of Dengue and Zika virus strains used in this study for infections in rhesus macaques. Animal numbers in experimental groups are small. Authors have stated some of the limitations of the study.

We thank the reviewer for this suggestion and have added a section to the discussion regarding the pathogenic potential of both the DENV and ZIKV isolates used in this study. While initial evidence from multiple studies across different primate centers suggested that about 1 in 4 pregnancies ended with adverse fetal outcomes (including animals exposed to ZIKV-PRVABC59), the data generated since this study was initiated suggests that the specific model used here generates adverse fetal outcomes in approximately 1 in 7 pregnancies, similar to what has been established in humans {29967348}. While we do not know how a different ZIKV isolate may have replicated, the use of a low-pathogenicity isolate may actually be an ideal choice for this study as it would have the potential to show enhancement as a result of pre-existing DENV immunity.

We agree that the small number of animals enrolled in the experiment is a limitation of the study. Please see our first comment to Reviewer #1 in the Methods section above.

Editorial and Data Presentation Modifications?
Use this section for editorial suggestions as well as relatively minor modifications of existing data that would enhance clarity. If the only modifications needed are minor and/or editorial, you may wish to recommend "Minor Revision" or "Accept".

Reviewer #1: I would like to suggest some changes in the manuscript that may aid in the improvement of the article quality. I suggest that the authors add an explanation on why the 45th gestational day was chosen for ZIKV challenges (line 136). In addition, I suggest that the paragraph from the line 154 to the line 161 be summarized or even excluded and that the information contained in that part of the text be placed in the introduction section and/or in the discussion section of the manuscript.

We have added a rationale for using gestation day 45 as the inoculation date.

We have moved this paragraph to the introduction.

Reviewer #2: (No Response)

Reviewer #3: (No Response)

Reviewer #4: No data are presented on the levels of inflammatory cytokines or monocyte activation during viral infections.

We agree that a more comprehensive study addressing the antigenic/immune interactions between DENV and ZIKV is an important one, but this is beyond the scope of the current project. Please see our response to Reviewer #3 in the Conclusions section above.

Summary and General Comments

Use this section to provide overall comments, discuss strengths/weaknesses of the study, novelty, significance, general execution and scholarship. You may also include additional comments for the author, including concerns about dual publication, research ethics, or publication ethics. If requesting major revision, please articulate the new experiments that are needed.

Reviewer #1: The work by Crooks et al. entitled “Prior dengue immunity enhances Zika virus infection of the maternal-fetal interface in rhesus macaques” evaluated the impact of prior DENV immunity over the infection of maternal-fetal interface tissues with ZIKV in macaques. This is the first study to assess the aforementioned relationship in a translational non-human primate model. The authors observed a significantly greater burden of ZIKV RNA in the chorionic plate in DENV-immune macaques as compared to DENV-naïve macaques as well as an association between prolonged viremia and the presence of ZIKV vRNA in the maternal-fetal interface. Although it is an interesting, unprecedented study, it has some important limitations. A small number of animals was enrolled. Only one DENV serotype (DENV-2) was tested. In addition, differences between the periods from DENV inoculation to ZIKV challenge in each individual were observed (up to 35 days). Moreover, since previous evidence suggest that the elapsed time from the DENV exposure to the ZIKV infection may influence in the risk of congenital Zika syndrome (Carvalho MS et al. Association of past dengue 727 fever epidemics with the risk of Zika microcephaly at the population level in Brazil. Sci Rep. 2020;10:1752), the comparison between groups of macaques with different elapsed times between DENV and ZIKA infections would be useful.

We thank the reviewer for their careful review of this manuscript. We have addressed each of these comments in the Methods and Results section above and updated the manuscript accordingly.

Reviewer #2: The authors conducted a study to assess if the pre-existing immunity to dengue virus could enhance Zika virus disease by the antibody-dependent enhancement phenomenon.

The proposed theme is of great relevance to understanding the risks of congenital malformation in children whose mothers have been infected with Zika virus, especially in areas endemic to

other flaviviruses. This hypothesis is based on studies that suggest this phenomenon's occurrence in vitro and animal models, but which has been widely contested as a hypothesis to justify the worsening of secondary cases of Dengue virus infection in humans.

The writing of the article is excellent and objective. The experiments were carried out with sophisticated methods and techniques and with high technical rigor. The data generated has been correctly analyzed and appears to be robust.

The results suggest that the pre-existing DENV immunity does not cause fetal abnormalities or even alter the Zika virus replication in maternal plasma.
Therefore, I am in favor of publishing the article in its present form.

We thank the reviewer for their thoughtful comments and critique of the manuscript.

Reviewer #3: This study focuses on the important and relevant topic of the impact of pre-existing dengue virus immunity on Zika virus infection in pregnant individuals using non-human primate models of infection. While the questions asked are significant and of high interest, the studies are incomplete and experiments too preliminary to justify conclusions.

We are grateful to the reviewer for their careful review of the manuscript. We have responded to the comments above and we believe that this has significantly strengthened the manuscript.

Reviewer #4: In this manuscript, Authors report that prior dengue immunity enhances Zika virus infection of the maternal-fetal interface in rhesus macaques. This study has examined the impact of pre-existing dengue virus immunity on Zika virus infection and pathogenesis during pregnancy in the rhesus macaque model. Authors found that prior DENV-2 exposure enhanced ZIKV infection of maternal-fetal interface tissues in macaques. However, pre-existing DENV immunity had no detectable impact on ZIKV replication kinetics in maternal plasma, and all pregnancies progressed to term without adverse outcomes or gross fetal abnormalities detectable at delivery.

Coinfections with dengue and Zika viruses have been reported in endemic regions. Therefore, it is important to understand the impact of re-existing immunity to any of these viruses on the outcomes of co-infections. It is of special interest to determine the impact of co-infections during pregnancy and on fetal growth and health. The manuscript reports that preexisting immunity to DENV leads to increased Zika virus infection in placenta. However, this did not lead to increased fetal abnormalities. Findings in this manuscript are very informative.

Data in Figure 2 demonstrate that pre-existing DENV-2 immunity did not increase or alter ZIKV replication kinetics during gestation. These data are consistent with previous reports of DENV and ZIKV co-infections in humans and non-human primates which did not result in increased

viral replication or enhancement of disease pathogenesis. Findings in this manuscript show that pre-existing DENV immunity did not dampen or protect against ZIKV infection.

Antibodies cross-reactive with ZIKV were found in DENV-immune animals. However, that did not interfere with the magnitude of antibody response to ZIKV. These findings are important since they showed that pre-existing cross-reactive DENV antibodies did not impair host immune response to ZIKV. Samples were not analyzed for several time points (due to COVID19 issues).

There was no evidence of vertical transmission of ZIKV in infants. QRT-PCR data are presented to show low levels of ZIKV RNA in placental samples. Although the assay may detect low levels of viral RNA in some of the animals from both groups (DENV-immune and DENV-naïve), data are not convincing to sufficiently demonstrate that there was enhanced infection at maternal-fetal interface. No infectious virus was recovered. Additionally, viral RNA levels are too low and the animal numbers per each group are not sufficient.

We thank the reviewers for this comment. Since there was a significant difference in only one tissue type, we have revised the manuscript to state that there was a higher burden in the chorionic villi rather than "enhanced infection." Additionally, we have revised the title of the manuscript to "Previous exposure to dengue virus is associated with increased Zika virus burden at the maternal-fetal interface in rhesus macaques"

These viral infections are known to induce inflammatory cytokine expression and monocyte activation. Considering the minimal impact of viral infections on the animals in this study, assessment of these parameters could determine whether the viral infections had any substantial effect on the host.

We agree that this is worthy of follow up, but it is beyond the scope of this study. Our overall goal was to assess pregnancy-specific outcomes. Importantly, pregnancies with in-utero ZIKV exposures can result in adverse fetal outcomes even if maternal exposure resulted in asymptomatic infection. Subsequent studies informed by the findings of this study will be needed to carefully dissect the immunological mechanisms underlying the effects we observed here.

There was no demonstrable placental pathology due to ZIKV infection in the present study. This raises the question about the pathogenic potential of the viral strains used to infect animals in the study. Authors need to comment on this issue.

We have included additional details on virus isolate pathogenicity in the discussion. Please see our response in the conclusions section above.
